# The Domestic Pet

**Published:** 1912-08

**Authors:** 


### The Domestic Pet

IT cannot be denied that domestication of animals has played a very important part in the civilization of the human race. The economic value of draft, milch, wool bearing and meat yielding animals, kept in captivity, trained to assist man in his labors or available as needed to supply his wants, requires no argument. It is not generally realized that the relative abundance of such animals in the eastern hemisphere and their almost entire absence in the western, is, without any other factor, sufficient to account for the different rates of progress of man in the old and the new world. With the exception of the llama, a domesticated huanaco, the paco, the turkey and certain central American fowls and rabbits, there were no domesticated animals in America prior to its discovery by Europeans. The llama was merely a pack animal, able to carry not much more than 50 pounds, far inferior to the camel or dromedary in all ways and, not being able to survive long in low altitudes, it could not have been utilized as a draft animal. The paco is far inferior to the sheep, as a supply of clothing. It is said that no animal indigenous to the new world would produce milk in captivity, and it is doubtful if the buffalo, deer and goats of the new world were susceptible of domestication.

These statements must be qualified by excepting the far north of America, where certain deer and dogs or domesticated wolves, played a useful role as in eastern arctic regions.

They must also be qualified, if we employ the word *domesticated* in the minor sense of *pet* animals. Various birds, and dogs were more or less domesticated by various Indian tribes; so, too, captive bears and other wild animals were rather freqeuent sources of amusement. The dog, in the absence of herds and flocks of more useful animals, obviously could not fulfill his old world function of a shepherd's assistant; in a roadless country, he could not be used as a draft animal to any extent except when a crust had formed on snow; there being for the most part, little private property and little personal dishonesty, he was of slight value as a watch dog, though he might give an alarm when a village was attacked at night. It does not even appear that the Indian trained the dog to any great degree as a help to the hunter.

But; quite apart from the main economic value of domestic animals so conspicuous in the old world, and whose absence was so conspicuous in the new world, the mere fact of domesticating, disciplining and controlling and looking after the welfare of lower animals, helped mightily to civilize man, to teach him self control and self denial, and to develop the sense of responsibility. Every instance of cruelty and neglect, brought him loss and even suffering, and thus taught the lesson of mercy and prudence. To a limited degree, therefore, the Indian enjoyed the civilizing influence of domesticated animals, in common with our own ancestors and other natives of the eastern continent.

The realization of the educative, as contrasted with the economic value of association with the lower animals, survives in the old fashioned belief that it is a good plan to bring up children with cats and dogs and in the persistence of domestic pets of no practical use whatever.

But, with the increasing concentration of population and the growing appreciation of applied bacteriology, we are, at least in cities, coming very near the point at which the direct relations of man and lower animals must be discontinued. Already, in most large cities, there is a restriction or even prohibition, more or less strictly enforced, of the keeping of any practically useful animal or fowl. Dogs are usually subject to capture and destruction unless tagged. Cats are notoriously less perfectly domesticated and no practical system either of license or of systematic destruction has been evolved. Other small animals, birds, etc., are ignored.

The dog laws are based on a somewhat exagerated notion of hydrophobia and certain misconceptions as to its times of prevalence. It is almost certain that many cases of so-called hydrophobia are tetanus, non-specific sepsis or hysteria. Some extremists indeed deny that there is such a disease as rabies. Ecchinococcus disease, fortunately still rare in America, entozoa, ectozoa as carriers of septic germs, tuberculosis, diphtheria and exenthemata conveyed adventitiously in the hair, are, quantitatively, much more important dangers from the lower animals than rabies. In fact, it may be said that any infection to which man is liable, is apt to be disseminated by domestic pets, either through their own infection, or accidentally. With the hygiene of the animal impaired by confinement, and the opportunities for conveyance magnified by density of population, it becomes more and more questionable whether the educative advantages, companionship and interest to be credited to the dog—or to any other domestic pet—counterbalance the physical risk.

Dwellers in the country or even in fairly large cities, can scarcely realize the objection to the dog in the really great cities, solely on account of his evacuations. It is true that those of the horse are quantitatively much more important but, save for the occasional presence of the tetanus bacillus which, to become infective, requires introduction beneath the skin and exclusion of air, horse manure is only slightly objectionable and scarcely an item of hygienic anxiety except regarding the breeding of flies. The practical value of the horse still far outweighs the disadvantage of his excrement. Indeed, the very bulk of the latter necessitates street cleaning which would be otherwise desirable but would probably be neglected, while its physical properties facilitate the collection of other, more dangerous dirt, much as tea leaves facilitate domestic sweeping.

The deposits of the dog are more offensive and are almost as definitely limited to sidewalks, etc., as those of the horse are to the street. In closely built cities, there are often for miles, no grass plots or other places where the dog can deposit his filth. Even the roofs, which in recent times have become the recognized offsets for the lack of yards in great cities, are in New York and analogous European cities, highly contaminated. Probably bacteriuria, at least involving any disease germ, is rare in dogs. But unless highly trained, every dog has two dominant impulses, first to detect every perpendicular object upon which any other dog has urinated and to follow suit; secondly to start as many other spots of the same kind as possible. These impulses are so well marked and so peculiar as to suggest a sort of undeveloped religious principle. Even if hygienically significant canine bacteriuria occurs once in a million cases, the chance of infection is, by this habit, multiplied by millions. Eventually, the minute chance of danger becomes actual.

The presence of dogs—not to mention cats, etc.—in large cities, also has certain sociologic bearings. There is no mental uplift at the common sight of a girl tugging at the leash of a large dog practicing the elemental principles mentioned, or others which need not be mentioned, or deliberately urging him to the daily hygienic task. There is even less uplift when she is subjected to the comments of a crowd of unclean-minded little boys and grown hoodlums. Is it too far fetched to suggest that the dog in dense centers of population has something to do with developing indelicacy and, ultimately immorality?

